# Strategic leadership capacity building for Sub-Saharan African health systems and public health governance: a multi-country assessment of essential competencies and optimal design for a Pan African DrPH

**DOI:** 10.1093/heapol/czx162

**Published:** 2018-07-08

**Authors:** Irene Akua Agyepong, Uta Lehmann, Elizeus Rutembemberwa, Suzanne M Babich, Edith Frimpong, Aku Kwamie, Jill Olivier, Gina Teddy, Boroto Hwabamungu, Lucy Gilson

**Affiliations:** 1 Ghana Health Service, Division of Research and Development, Dodowa Health Research Center, Dodowa, Greater Accra, Ghana; 2 School of Public Health, University of the Western Cape, Robert Sobukwe Road, Cape Town, Bellville, South Africa; 3 Department of Health Policy Planning and Management, Makerere University, School of Public Health, Uganda, East Africa; 4 Indiana University, Richard M. Fairbanks School of Public Health Health Sciences Building (RG), 1050 Wishard Blvd, 6th Floor, Indianapolis, IN, USA; 5 School of Public Health and Family Medicine, Anzio Road, Observatory, Cape Town, South Africa; 6 Ghana Institute of Management and Public Administration, Center for Health Systems and Policy Research (CHESPOR), Achimota, Accra, Ghana; 7 Department of Global Health and Development, London School of Hygiene and Tropical Medicine, Keppel Street, London, UK) and; 8 University of Ghana, School of Public Health, Department of Health Policy, Planning and Management, Legon, Accra, Ghana

**Keywords:** Capacity building, governance, health systems, needs assessment, public health, strategic leadership, sub-Saharan Africa

## Abstract

Leadership capacity needs development and nurturing at all levels for strong health systems governance and improved outcomes. The Doctor of Public Health (DrPH) is a professional, interdisciplinary terminal degree focused on strategic leadership capacity building. The concept is not new and there are several programmes globally–but none within Africa, despite its urgent need for strong strategic leadership in health. To address this gap, a consortium of institutions in Sub-Saharan Africa, UK and North America have embarked on a collaboration to develop and implement a pan-African DrPH with support from the Rockefeller Foundation. This paper presents findings of research to verify relevance, identify competencies and support programme design and customization. A mixed methods cross sectional multi-country study was conducted in Ghana, South Africa and Uganda. Data collection involved a non-exhaustive desk review, 34 key informant (KI) interviews with past and present health sector leaders and a questionnaire with closed and open ended items administered to 271 potential DrPH trainees. Most study participants saw the concept of a pan-African DrPH as relevant and timely. Strategic leadership competencies identified by KI included providing vision and inspiration for the organization, core personal values and character qualities such as integrity and trustworthiness, skills in adapting to situations and context and creating and maintaining effective change and systems. There was consensus that programme design should emphasize learning by doing and application of theory to professional practice. Short residential periods for peer-to-peer and peer-to-facilitator engagement and learning, interspaced with facilitated workplace based learning, including coaching and mentoring, was the preferred model for programme implementation. The introduction of a pan-African DrPH with a focus on strategic leadership is relevant and timely. Core competencies, optimal design and customization for the sub-Saharan African context has broad consensus in the study setting.

**Key Messages**

- The target group for a pan-African DrPH with a focus on strategic leadership should be people who already have experience in leadership at the operational level and higher in the health sector.
- The intellectual rigor of terminal (doctoral level) training should be applied in the programme but with emphasis on practice, application, innovation, leadership and management.
- A programme approach that involves a mix of short periods of campus residence with longer periods that enable workplace based learning, facilitated by coaching and mentoring is identified as the most relevant capacity development approach. The details and optimal mix of residential periods and workplace based learning need to be finalized in context.
- Learning must be practice-based with an emphasis on application of theory rather than being didactic and predominantly theory driven.

## Introduction

Despite improvements in health outcomes, much of Sub-Saharan Africa continues to lag behind global averages (GBD 2010; United Nations 2013). The reasons are multiple and complex. Beyond direct causes of morbidity and mortality, such as communicable and non communicable diseases, wider environmental and contextual socio-economic determinants of health as well as the strength or resilience of health systems affects these outcomes. Health systems include all the resources, actors and institutions related to the financing, regulation and provision of health actions; where health actions are any set of activities whose primary intent is to improve or maintain health (Murray and Frenk 2000). Health systems are part of the core concern of public health. Drawing on the Institutes of Medicine report, by public health we refer to a discipline whose mission is ‘assuring the conditions in which people can be healthy’ (IOM 1988). Public health seeks to achieve this mission by preforming the three related core functions of assessment (information collection, analysis and dissemination), assurance (implementation) and policy development (decision making).

**Figure 1:**
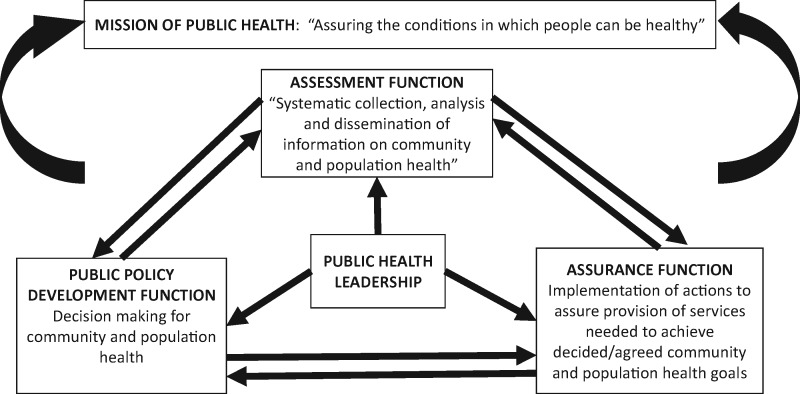
Public Health mission, functions and leadership

People and their power in deciding, shaping and responding to change are central to health systems; and leadership, an essential component of health system governance (WHO 2007), is an important influence on the performance of the core functions of public health as summarized in Figure 1. At the 15th assembly of heads of state and government of the African region, leadership and governance challenges were among several reasons cited for the persisting high maternal mortality ratios in sub-Saharan Africa. (Sambo *et al*. 2011). Similarly, a literature review of health system barriers that could explain why despite a decade of implementation of intermittent preventive treatment for malaria in pregnancy, utilization of the intervention was so low, identified poor leadership and governance among contributors to the situation (Thiam *et al*. 2013). Ijsselmuiden *et al*. (2007), meanwhile, in a mapping of Africa’s advanced public health education capacity broadly define public health professionals as ‘those responsible for providing leadership and expert knowledge to health systems at district, provincial, national and international levels to manage the health of the public.’ They noted that in this, as in many other areas, Africa is relatively deprived compared to the rest of the world.

What exactly do we mean by leadership? Within organizational literature (e.g. Nahavandi 2000; Crossan *et al*. 2008; Pearce *et al*. 2014), including the limited African scholarship e.g. Masango (2003), leadership is commonly conceived as: a group phenomenon (entailing leaders and followers), an influence process (where leaders exerts influence on followers) and a process that is inspirational and goal-oriented. Cacioppe (1998), cites Kotter (1990) in defining leadership as: ‘the creation of a vision and strategic direction for an organization, the communication of that vision to the people and customers of the organization and also involves inspiring, motivating and aligning people and the organization to achieve this vision.’ The International Hospital Federation (2015) similarly defines leadership as: ‘the ability to inspire individual and organizational excellence, create a shared vision and successfully manage change to attain an organizations strategic ends and successful performance.’ Leadership is an essential part of the function of governance within health systems.

We draw our definition of strategic leadership from Adair’s (2005) conceptualization of three levels of leadership. The team level involves leadership of a small group focused on a given task or tasks. The operational level involves leadership of several team leaders. The strategic level (also referred to as the upper echelon) involves leadership of a whole organization or major division of an organization with several operational leaders under one’s direction. Strategic leadership could be at national, sub-national or international agency level depending on the complexity and size of the organization. It demands the abilities to make decisions, develop organizational structures, processes and controls and manage multiple constituencies. It also require abilities to motivate others by creating and communicating a future vision, and to develop an environment that enables widespread distributed and participatory leadership (House and Aditya 1997; Hickman 1998; Ireland and Hitt 1999; Bolden 2011).

How can strategic and participatory leadership, as an orientation and capacity be developed within African health systems? (Chunharas and Davies 2016; AHPSR 2016) Since the Alma Ata Declaration of 1978 much effort has been put into capacity building for health system managers and leaders in sub-Saharan Africa, with a dominant focus on operational leadership and, in particular, district managers (Conn *et al*. 1996; Kwamie *et al*. 2015). The most common degree, a *Master of Public Health*, has gained currency and become a wide-spread pre-requisite for public health practice. Doctoral training has been advanced less commonly, and where it is offered in Africa has traditionally been the Doctor of Philosophy (PhD) degree, structured and targeted at those who wish to pursue predominantly academic and research career pathways.

Internationally, however, another terminal degree exists for public health professionals: the Doctor of Public Health (DrPH). The DrPH is targeted at people who want to ‘use the critical thinking, analytic and synthetic skills of a terminal degree predominantly in professional public health leadership and practice and aims to prepare individuals for evidence-based public health leadership, including practice oriented research and field-based roles’ (ASPPH 2009). Although DrPH programmes are available in the US, Europe, and Asia, there was no DrPH programme available in Africa at the start of this work (Agyepong *et al*. 2015).

To fill this vacuum, a collaborative initiative between academic and practice institutions in Sub-Saharan Africa (University of Ghana, Ghana Health Service, University of Cape Town, University of the Western Cape, Makerere University); the United Kingdom (London School of Hygiene and Tropical Medicine) and North America (University of North Carolina at Chapel Hill, Indiana University); was established with support from the Rockefeller Foundation to develop and implement a pan-African DrPH. The partner institutions were purposively and strategically selected based on their capacity and experience; and also to span East, West and Southern Africa. The vision of the consortium was to support and nurture the development of strategic leadership within the health sectors of sub-Saharan African; towards improved health outcomes.

As a first step towards programme development, we undertook this assessment with two related objectives. The first was to explore more widely and empirically perceptions of the need for a pan-African DrPH, with a focus on strategic leadership. The second was to generate evidence to inform the customization of programme design to context, in terms of competencies, content and mode of delivery.

## Methods

The study was a multiple case study of Ghana, South Africa and Uganda. In each case, the unit of analysis was the same; and defined as perceptions of the need for a pan-African DrPH focused on strategic leadership capacity, relevant competencies, content and mode of delivery. Cross sectional mixed methods of data collection were undertaken in all three countries in 2014/15. Data collection involved a non-exhaustive desk review of grey and published literature, key informant (KI) in-depth interviews, administration of a semi-structured questionnaire with closed and open ended items; and a stakeholder validation workshop in Ghana. Table 1 presents key contextual variables across countries and Table 2 summarizes data collection by country. Variation across countries reflects differences in opportunities for data collection and validation.

**Table 1:** Data collection by country

Data collection method	Number participants or sessions per country			
	Ghana	South Africa	Uganda	Total
KI in-depth interviews	12	4	18	34
Semi-structured questionnaire	78	111	82	271
Stakeholder validation workshop	1	0	0	1

**Table 2:** Brief context of Uganda, Ghana and South Africa the study sites

	Uganda (East Africa)	Ghana (West Africa)	South Africa (South Africa)
GNI per capita (2014) (USD)^1^^,^^a^	670	1590	6800
U5MR (2014)^2^	55	62	41
Physicians/1000 pop^b^	0.117 (2005)	0.11 (2008)	0.758 (2011)
Nursing and midwifery personnel/1000 pop	1.306 (2005)	0.974 (2008)	4.72 (2011)
Governance (Macro)	Multi-party democracy with no fixed term limits for president. Elections every 4 years. Current president transitioned from military leader to elected leader and has won every election since (over 30 years).	Multi-party with maximum of two 4 year terms for the president. Elections every 4 years since 1992. Power rotates between two political parties who claim social democrat (NDC) and liberal democrat (NPP) ideology.	Multi-party with maximum of two 4 year terms for the president. Elections every 4 years. Single party ANC has won every election since apartheid was dismantled in 1994. President has maximum of two 4 year terms.
Population (Pop.)	34.9 million in 2014^c^	25 million in 2010 census^d^	54 million in 2014^e^
Pan African DrPH Partner Institutions	Makerere University	University of Ghana (UG). Ghana Health Service (GHS).	University of Cape Town (UCT). University of Western Cape (UWC).

1. Atlas method of conversion.

2. Under-five mortality rate is the probability per 1,000 live births that a newborn baby will die before reaching age five, if subject to age-specific mortality rates of the specified year.

a http://data.worldbank.org/indicator/NY.GNP.PCAP.CD Downloaded 15 June 2016.

b http://apps.who.int/gho/data/node.main.A1444. Downloaded 15/6/16.

c UBOS, National Population and Housing Census. 2014.

d Ghana Statistical Services. 2010 Population and Housing Census. Summary report of final results. May 2012.

e http://www.statssa.gov.za/publications/P0302/P03022014.pdf Downloaded 16/6/16.

### Document review

Documents reviewed included peer-review articles, books and grey literature. We additionally drew on an earlier review of international DrPH programme objectives, content and experiences (Agyepong *et al*. 2015).

### KI interviews

KI in-depth interviews lasted between 30 and 60 mins. They were semi-structured conversations in which a topic guide was used to elicit respondents’ own views on leadership competencies as well as appropriate approaches to leadership development in their context. Topic areas included: past and present personal leadership roles and experiences in the health sector, and perceptions of the key competencies individuals in strategic leadership in the health sector need. We also explored perceptions of how these competencies are best acquired, what should be looked for in selecting strategic leaders in health and what should go into the training and preparation of such leaders. We informed KI about the idea of the Pan African DrPH and asked for their opinion and suggestions on program design in terms of content, structure and process. At the end of the interview, we presented a summary list of the domains in the ASPPH competencies and asked KI to comment on their relevance to strategic leadership capacity building in their context.

Twelve KI interviews were conducted in Ghana, four in South Africa and eighteen in Uganda. KIs were people previously or currently working in strategic and operational level leadership positions. They were purposively selected through knowledge of the country health sectors and a snowballing approach where recommendations from those already interviewed, informed further interviews. They held or had held positions such as national and sub-national directors and deputy directors in ministries of health or national health services, NGO; chairpersons of national boards or institutes, and University professors in the position of head of a school, centre, college or provost. Table 3 summarizes background of KI by country. The majority being male (82%) and having a medical professional background reflects observations of the current demographic and professional dominance of strategic health sector leadership in the study settings.

**Table 3:** Selected background characteristics of KIs

	Country				
	Ghana	South Africa	Uganda	Total	% of total
Number interviewed	12	4	18	34	
Sex					
Male	11	3	14	28	82%
Female	1	1	4	6	18%
Professional background					
Physician (Non-public health)	1	1	1	4	12%
Physician (Public Health)	7	1	15	23	68%
Pharmacist	3	1	––	4	12%
Social scientist	1	––	2	3	9%
Psychologist	––	1	––	1	0%
Predominant sector of work and leadership experience					
Public	9	3	15	27	79%
Private	2	––	3	5	15%
Both public and private	1	1	––	2	6%
Highest health system agency level of leadership					
National	10	2	13	25	74%
Sub-national	2	2	5	9	26%

**Table 4:** Validation of (ASPPH 2009) competencies [Upon graduation a DRPH trainee should be able to: (Yes = Agree; No = Disagree; DK = Don’t Know)]

	Ghana					South Africa					Uganda					Combined				
	Yes	No	DK	Total	% Agree	Yes	No	DK	Total	% Agree	Yes	No	DK	Total	% Agree	Yes	No	DK	Total	% Agree
Analyse the impact of legislation, judicial opinions, regulations and policies on population health	78	0	0	78	100%	107	1	3	111	96%	80	2	0	82	98%	265	3	3	271	98%
Establish goals, timelines, funding alternatives and strategies for influencing policy initiatives	77	1	0	78	99%	104	1	4	109	95%	79	0	3	82	96%	260	2	7	269	97%
Design action plans for building public and political support for programs and policies	77	1	0	78	99%	100	3	5	108	93%	80	1	1	82	98%	257	5	6	268	96%
Develop evidence based strategies for changing health law and policy	76	1	1	78	97%	104	0	4	108	96%	81	1	0	82	99%	261	2	5	268	97%
Understand and utilize international diplomacy and negotiation skills for the promotion of health	68	3	5	76	89%	97	2	10	109	89%	77	3	2	82	94%	242	8	17	267	91%
Discuss the inter-relationships between health communication and marketing	77	1	0	78	99%	89	4	8	101	88%	72	3	7	82	88%	238	8	15	261	91%
Explain communication program proposals and evaluations to lay, professional and policy audiences	78	0	0	78	100%	91	0	10	101	90%	73	3	6	82	89%	242	3	16	261	93%
Employ evidence based communication program models for disseminating research and evaluation outcomes	77	1	0	78	99%	95	1	4	100	95%	80	1	1	82	98%	252	3	5	260	97%
Guide an organization in setting communication goals, objectives and priorities, including risk communication during epidemics/pandemics	77	1	0	78	99%	93	0	7	100	93%	76	3	3	82	93%	246	4	10	260	95%
Create informational and persuasive communications	76	1	1	78	97%	90	3	7	100	90%	68	10	4	82	83%	234	14	12	260	90%
Participate actively and meaningfully in international health discussions and fora	78	0	0	78	100%	96	0	3	99	97%	75	3	4	82	91%	249	3	7	259	96%
Integrate health literacy concepts in all communication and marketing initiatives	75	1	2	78	96%	88	3	8	99	89%	66	5	11	82	80%	229	9	21	259	88%
Develop formative and outcome evaluation plans for communication and marketing efforts	72	5	0	77	94%	89	2	9	100	89%	69	3	10	82	84%	230	10	19	259	89%
Prepare dissemination plans for communication programs and evaluations	78	0	0	78	100%	92	1	5	98	94%	70	5	6	81	86%	240	6	11	257	93%
Propose recommendations for improving communication processes	76	2	0	78	97%	96	1	5	100	96%	75	3	3	81	93%	247	6	8	259	95%
Develop collaborative partnerships with communities, policy makers and other relevant groups, esp MDAs and civil society organizations	77	1	0	78	99%	84	1	8	93	90%	80	0	2	82	96%	241	2	10	253	95%
Engage communities in creating evidence based, culturally competent programs	76	2	0	78	97%	88	1	4	93	95%	69	8	4	81	85%	233	11	8	252	92%
Conduct community based participatory intervention and research projects	76	2	0	78	97%	89	3	1	93	96%	75	5	1	82	93%	240	10	2	253	95%
Design action plans for enhancing community and population based health	76	2	0	78	97%	92	1	0	93	99%	74	3	5	82	90%	242	6	5	253	96%
Assess cultural, environmental and social justice influences on the health of communities	77	1	0	78	99%	88	2	3	93	95%	75	3	4	82	91%	240	6	7	253	95%
Implement culturally and linguistically appropriate programs, services and research	74	1	3	78	95%	87	3	4	94	93%	68	9	5	82	83%	229	13	12	254	90%
Apply theoretical and evidence based perspectives from multiple disciplines in the design and implementation of programs, policies and systems	77	1	0	78	99%	91	0	0	91	100%	79	1	2	82	96%	247	2	2	251	98%
Interpret quantitative and qualitative data following current scientific standards	78	0	0	78	100%	89	0	1	90	90%	79	2	1	82	96%	246	2	2	250	98%
Design needs and resource assessments for communities and populations	76	1	1	78	97%	86	0	5	91	95%	76	4	2	82	93%	238	5	8	251	95%
Develop health surveillance systems to monitor population health, health equity and public health services	77	1	0	78	99%	83	4	2	89	93%	75	6	1	82	91%	235	11	3	249	94%
Synthesize information from multiple sources for research and practice	78	0	0	78	100%	86	1	3	90	96%	81	0	0	81	100%	245	1	3	249	98%
Evaluate the performance and impact of health programs, policies and systems	78	0	0	78	100%	87	1	1	89	98%	81	1	0	82	99%	246	2	1	249	99%
Weigh risks, benefits and unintended consequences of research and practice	73	5	0	78	94%	87	1	2	90	97%	76	2	3	81	93%	236	8	5	249	95%
Communicate an organization’s mission, shared vision and values to stakeholders	76	1	1	78	97%	88	1	2	91	97%	75	5	2	82	91%	239	7	5	251	95%
Develop teams for implementing health initiatives	77	1	0	78	99%	89	1	1	91	98%	74	6	2	82	90%	240	8	3	251	96%
Collaborate with diverse groups	75	2	1	78	96%	88	0	2	90	98%	70	9	3	82	85%	233	11	6	250	93%
Influence others to achieve high standards of performance and accountability	76	0	2	78	97%	86	0	3	89	97%	75	6	1	82	91%	237	6	6	249	95%
Guide organizational decision making and planning based on internal and external environmental research	77	1	0	78	99%	89	0	2	91	98%	79	1	2	82	96%	245	2	4	251	98%
Prepare professional plans incorporating lifelong learning, mentoring and continued career progression strategies	77	0	1	78	99%	84	1	5	90	93%	78	1	3	82	95%	239	2	9	250	96%
Create a shared vision	75	2	1	78	96%	85	2	4	91	93%	68	9	5	82	83%	228	13	10	251	91%
Develop capacity building strategies at the individual, organizational and community level	76	2	0	78	97%	88	2	1	91	97%	77	5	0	82	94%	241	9	1	251	96%
Demonstrate a commitment to personal and professional values	76	1	1	78	97%	89	0	2	91	98%	73	6	2	81	90%	238	7	5	250	95%
Implement strategic planning processes	75	2	1	78	96%	87	1	1	89	98%	80	2	0	82	96%	242	5	2	249	97%
Apply principles of human resource management	77	1	0	78	99%	89	1	0	90	99%	75	7	0	82	91%	241	9	0	250	96%
Use informatics principles in the design and implementation of information systems	71	3	4	78	91%	83	2	5	90	92%	66	7	9	82	80%	220	12	18	250	88%
Align policies and procedures with regulatory and statutory requirements	72	3	3	78	92%	85	0	4	89	96%	77	4	0	81	95%	234	7	7	248	94%
Deploy quality improvement methods	76	1	1	78	97%	87	2	1	90	97%	75	5	2	82	91%	238	8	4	250	95%
Organize the work environment with defined lines of responsibility, authority, communication and governance	76	1	1	78	97%	84	0	6	90	93%	73	5	4	82	89%	233	6	11	250	93%
Develop financial and business plans for health programs and services	74	1	3	78	95%	84	2	4	90	93%	75	7	0	82	91%	233	10	7	250	93%
Establish a network of relationships, including internal and external collaborators	78	0	0	78	100%	87	0	0	87	100%	75	4	2	81	93%	240	4	2	246	98%
Evaluate organizational performance in relation to strategic and defined goals	77	1	0	78	99%	90	0	0	90	100%	75	4	1	80	94%	242	5	1	248	98%
Identify and Manage potential conflicts of interest encountered by practitioners, researchers and organizations	76	1	1	78	97%	83	2	3	88	94%	73	7	2	82	89%	232	10	6	248	94%
Differentiate among the administrative, legal, ethical and quality assurance dimensions of research and practice	77	0	1	78	99%	88	0	0	88	100%	73	6	3	82	89%	238	6	4	248	96%
Design strategies for resolving ethical concerns in research, law and regulations	72	4	2	78	92%	79	2	7	88	90%	70	9	2	81	86%	221	15	11	247	89%
Develop tools that protect the privacy of individuals and communities involved in health programs, policies and research	76	0	2	78	97%	82	2	3	87	94%	73	7	2	82	89%	231	9	7	247	94%
Prepare criteria for which the protection of the public welfare may transcend the right to individual autonomy	73	2	3	78	94%	73	0	13	86	85%	67	6	9	82	82%	213	8	25	246	87%
Assess ethical considerations in developing communications and promotional initiatives	77	1	0	78	99%	84	0	3	87	97%	76	5	1	82	93%	237	6	4	247	96%
Demonstrate cultural sensitivity in ethical discourse and analysis	77	0	1	78	99%	85	0	3	88	97%	72	5	2	79	91%	234	5	6	245	96%

All interviews were conducted face to face in English by the same senior member of the research team in Ghana (IAA) and South Africa (LG); and two experienced qualitative interviewers in Uganda. Additional to interviewer notes, interviews were tape recorded and transcribed. Thematic analysis was conducted to identify the main themes of need for the program, competencies, content and mode of delivery; and no particular competencies framework was imposed on the data.

### Semi-structured questionnaire

In the USA, the DrPH is almost the default, terminal degree in the field of Public Health. The USA Association of Schools and Programmes of Public Health (ASPPH) has developed DrPH competencies based on a rigorous, modified Delphi process (ASPPH 2009). We designed a questionnaire with closed and open ended items to validate their specific relevance to sub-Saharan Africa and the general relevance of the concept of a pan-African DrPH. These core domains are shown in Table 2, which also presents the results of the semi-structured interviews.

In all three countries, selection of respondents was purposive with the emphasis on getting opinions from a wide range of respondents in varying health sector settings rather than statistical generalizability. Interviewees were identified as willing and able to participate, and as potential DrPH candidates, i.e. currently working in a mid or senior level management and leadership position in the health sector, with a Master in Public Health or equivalent degree and several years of working experience.

In Ghana and Uganda, the survey was interviewer administered. Respondents in Ghana were from two adjacent regions, Greater Accra which is 90% urban with predominantly metropolitan and municipal districts and contains Accra the capital of Ghana; and Eastern, which has a 50–60% rural population, with some rural districts remote, deprived and difficult to access. In Uganda respondents were from the highly urbanized, capital Kampala and the nearby district of Mukono as well as a big town in the more rural Northern part of the country, Gulu. Response rates in Ghana and Uganda were 100%. In South Africa, the survey was administered electronically using a survey monkey tool, which generated a link that was emailed to participants. Alumni and current MPH students from the Universities of Cape Town and the Western Cape in senior and middle level management positions in the health sector were targeted. To encourage participation, respondents were reminded twice before the end of the deadline. In South Africa, a total of 111 responses were received from 400 emails inviting participation in the survey, giving a response rate of around 25% (a common response rate for a survey of this kind). Simple responses rate frequencies were calculated by question, for each country and compared across countries.

### Stakeholder validation workshop

In Ghana, a stakeholder validation workshop was held in April 2015 with 16 participants–of whom, 7 had participated in the KI interviews; 2 in the semi-structured interviews; 5 were people whom we had tried, but failed, to interview previously and 2 were from the University of Ghana School of Public Health. The draft findings and conclusions of the assessment, were presented for their feedback.

Finally, in a consortium partners meeting in Cape Town in February 2015, team members reviewed and synthesized the data from the different sources to develop a Pan African DrPH competencies and program outline.

## Results

### Is there a need for a Pan African DrPH with a focus on strategic leadership?

To answer our first question, we drew on results from the desk review of programs for strategic leadership training in the three countries as well the primary data. Though there were no DrPH programmes in sub Saharan Africa at the time of this study, there were several non-degree short courses and a few degree programmes with some leadership capacity building focus. They were usually of a generic nature rather than specifically targeted at the health sector. Examples of such programmes, rather than a comprehensive review, are provided below to illustrate.

The Ghana Institute of Management and Public Administration (GIMPA) a public self-financing University had an established executive Masters in Governance and Leadership and was well advanced in planning for a PhD in Governance, Leadership and Public Administration (http://www.gimpa.edu.gh). The Graduate School of Governance and Leadership (GSGL) of the Almond Institute, a private Christian institution of higher education affiliated with the Kwame Nkrumah University of Science and Technology (KNUST), had a BSc International Business Administration and Global Leadership.

In South Africa, the range of formally accredited programmes included post-graduate diploma and Masters level programmes offered by Business and Public Management Schools to a broad audience that included health managers. One KI noted, however, that business school courses do not ‘bring a coherent synthesis of tools relevant to public health, linked to the nuances of this context.’ The available health-specific university programmes were all at the postgraduate level. The oldest offering was the postgraduate Diploma in Health Management of the University of Cape Town, initiated in 1996 and formally known as the Oliver Tambo Fellowship Programme. The more recently initiated Albertina Sisulu Executive Leadership Programme in Health (ASELPH) came into existence in 2013, offered both a postgraduate Diploma in Health Systems Management and an MPH, and was supported by the Universities of Pretoria, Fort Hare and Harvard. Both programmes primarily targeted public sector health managers, and had close links with provincial and national departments of health.

In Uganda, Makerere University offered a 2 year non-degree awarding fellowship programme whose target audience were those who had a post-graduate degree in a health-related field such as Public Health, Medicine, Statistics, Journalism, Social Sciences, Demography or Information Technology, working in government or the private sector. The goal of the fellowship was to groom and nurture a cadre of transformative leader-managers who could think critically, have a high level of interdisciplinary thinking and effectively work with and through teams to lead and manage programmes. Makerere University Business school offered a Master of Science in Leadership and Governance. A Masters in Public Health Leadership, specifically targeted at people interested in improving maternal health, was offered by the Uganda Christian University, a private University.

High levels of support were expressed by KI for the concept of a Pan African DrPH program with a focus on strategic leadership. Several respondents urged implementation sooner rather than later.

*‘clearly there is a need’ KI South African.*

*‘this course is long overdue (…) When does the course start? I can’t wait!’ KI South Africa.*

*‘Use me as a foundation student (…) It will help’ KI Ghana.*

However, there were some dissenting voices. A few of the KI in-depth interview respondents in Uganda raised questions about the relevance of a terminal degree for professional Public Health practice.

**Figure 2:**
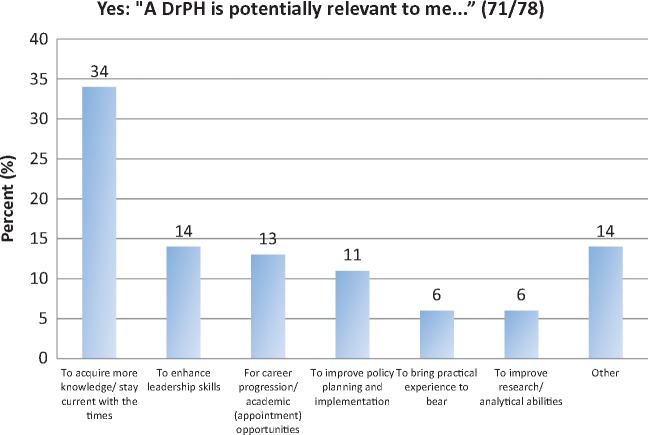
Reasons given by the 71 out of 78 respondents in Ghana who felt a DrPH was potentially relevant to them Note: Responses classified as ‘Other’ in figure 2 had one respondent in each of the following categories: accessing current technology, enhancing management skills, strengthening analytical skills, learning to better teach subordinates, increasing negotiation skills and for the prestige of the qualification.

In the Ghana semi-structured interviews for potential DrPH trainees; respondents were asked: ‘Do you think the concept of a professional doctoral level leadership training programme is potentially of relevance to you.’ They were also asked to explain their response. Seventy one out of seventy eight respondents (91%) said ‘yes’ (Figure 2); but eight said ‘no’, with reasons outlined in Box 1. Some of these responses are similar to the perceptions from a few of the Ugandan KI interviewees that a terminal degree is a personal ambition rather than needed to strengthen leadership practice.

### Competencies, content and mode of program delivery

To answer our second question, we drew on data from the KI in-depth interviews, the semi structured questionnaire interviews for mid level managers and the stakeholder validation workshop.

### KI perceptions

Perceptions of KI across all three countries on strategic leadership competencies needed at national and sub-national levels in their context can be grouped into seven competency clusters we labelled: (1) core personal values or self-mastery; (2) visioning, inspiring and leading change; (3) skills for interacting and working with people; (4) managing complexity and context including politics; (5) generating, analysing, synthesizing and using evidence; (6) managerial function skills and (7) technical skills of relevance to the professional area in which you lead. We briefly explain each cluster drawing on quotes from the KI interviews to illustrate.

#### Core personal values and character (self mastery)

Core personal values and integrity of character repeatedly came up as important for leadership. We use the term ‘self mastery’ to describe these competencies since they relate to the ability to lead oneself from within. Integrity or a wholeness and consistency in character, and the related concepts of trustworthiness and honesty were identified across interviews. Other characteristics mentioned as important and which we place in this cluster include the ability to adapt, to see leadership as hard work and service rather than just position and perks, meticulousness, people (including gender) sensitivity, confidence, personal stamina, resilience and fortitude, and professionalism and ethics in leadership practice.

‘…… your own personality itself it makes a big difference’ (KI Uganda).

‘(…) let me use the word core values (….). You must have the ability to discern what is wrong from what is right (…) You must be honest. You must be trustworthy. It sums up by the use of the term integrity’ (KI Ghana).

#### Visioning, inspiring, leading change

Visioning, inspiring and leading change as a leadership competency was a recurring theme. One South African KI distinguished management from leadership training, noting that leaders are: ‘people who deal with novel situations not encountered before, who can re-write scripts and enrich processes, and who must be able to deal with unexpected problems.’ In this regard, understanding systems as complex, policy as a process and accountability as about outcomes not compliance, as well as reflective practice, were identified as important.

The ability to vision, inspire people to share the vision and work towards its attainment by leading change was thought to require a vision rooted in the aspiration of the people and the organization, rather than a personality focused vision. It needed to be futuristic and yet take into account current realities. Linking back to the competency of self mastery, part of this competency was to put the interest of the organization before personal interests if any conflict of interest arises.

‘Ability to envision the future based on current realities’ (KI Ghana).

‘Requires, not just skills in day to day guiding; need to have leaders who can think ahead and translate experience into real progress, leaders who can paint broad strategic picture and goals to improve the system’ (KI South Africa).

‘So leadership is basically to give the team you are working with a general direction to achieve the objectives and goals you have set as a department (…) as a ministry or as a sector’ (KI Uganda).

##### Skills for interacting and working with people

As strategic leaders have to lead with and through people rather than doing everything themselves, skills for interacting, working with and managing people were judged important. This includes the ability to ensure an appropriate human resource mix, matching people and their skills to task requirements. The skill to work in, with and through teams, communication skills, conflict resolution, negotiation and listening were all mentioned as critical.

‘At this level it is about people relationships, engaging with people’ (KI Ghana).

‘Teams are really important–fundamental, and people management, key’ (KI South Africa).

‘(.,) convincing, (…) counseling, (…) defining information, putting together an agenda, building trust, building confidence and getting people round the table to agree and be able to commit to drive a program’ (KI Uganda).

Also included in this cluster are the ability and responsibility of the strategic leader for ensuring coaching and mentoring, to empower and develop people in the organization.

‘Your job is to coach and mentor your directors to harness their own competencies and do what they have to do. Practical skill of coaching and mentoring is fundamental’ (KI Ghana).

‘Team building because there is always team work, but we have to build a team before we can work with a team, so how do we build a team? I think this is a key question … you have to appreciate that everybody you are working with matters, from the sweeper if that is the lowest up to the highest person. So, you have to find a way of building a team and that’s where communication comes in’ (KI Uganda).

##### Navigating complexity and context, including politics

Context awareness and skills in navigating sub-national, national as well as international context and complexity, including the ability to adapt principles and programmes to context was identified in several interviews. It included the ability to work with a multiplicity of stakeholders and interests, across levels, sectors and disciplines; and with complex problems demanding creative problem solving.

‘The ability to contextualize local, national, international policies ideas and concepts into relevant local national policies. Cut and paste does not work’ (KI Ghana).

‘a lot of work at strategic level is a complex interplay between research, public and private interests’ (KI South Africa).

Included in this cluster are the ability to navigate institutional as well as micro and macro politics and the skills and knowledge to leverage the political system.

‘The other skill is how to manage politics. Any strategic leader would be working with people who have different interests; public health unlike medicine is the interface with medicine and politics because we don’t deal with individuals but we are dealing with groups ‘(KI Uganda).

Engaging politics was seen as ‘… .difficult but necessary’ (KI South Africa); and good relationships with political leaders and communities as well as advocacy were part of the essential skills involved.

‘So, you must know how to engage yourself with leaders that are not in health but impact on health greatly’ (KI Uganda).

We also placed in this cluster, suggestions and comments related to creating systems that work despite public sector challenges; understanding, navigating and tackling organizational cultures to create systems that work and manage change. This includes innovativeness; and the ability to adapt and engage and work with civil society groups and communities including their leaders.

‘Key capability is the ability to adapt. When confronted with a situation don't just throw your hands into the air but confront it and ask what is the adaptive capacity that I should adopt (…)’ (KI Ghana).

‘(…) tolerance to variation in operational decision-making–allowing local level variation (…) is really important for innovation, and development of scalable models’ (KI South Africa).

##### Generating, analysing, synthesizing and using evidence

This competencies cluster included understanding of and skills in research, specifically operational and implementation research in complex environments. Analytical skills including policy analysis and development skills, stakeholder analysis, the ability to be reflective as well as analytical, and to think critically and strategize drawing upon data are all included in this cluster.

Strategic leaders must have ‘(….) comfort to interrogate large data bases in informing decision making. They must understand the logic of decision making with data, making intelligent decisions’ (KI South Africa).

‘(…) you need also to have analytical skills and competences where you can be able to see and look at like where your performance is and be able to know where you want to go (…)’ (KI Uganda).

##### Managerial skills

Managerial function skills that came up repeatedly in KI interviews across all three countries included project management and implementation skills, planning and implementing within available resources, financial management, forecasting and budgeting, human resource management, performance monitoring and evaluation and crisis management skills.

##### Technical skills of relevance to the area in which you lead

Many respondents felt that without a strong, core understanding, it would be difficult for anyone to lead in a given field, or even to gain true respect from the professionals they lead. The leader did not have to be the leading technical expert in that field, but definitely needed enough understanding to appreciate the work of experts and be credible in their eyes.

‘(…) within the GHS (Ghana Health Service) I will insist on having technical in addition to managerial skills and capacity’ (KI Ghana).

‘knowing the field where you are operating from (…) unless you know the operation of the place where you are it becomes very difficult to lead’ (KI Uganda).

Relevance of ASPPH DrPH competencies to an African programme.

Responses to the semi-structured questionnaire administered to potential DrPH trainees on the relevance of the ASPPH DrPH competencies were overwhelmingly positive (Table 3).

Similarly KI responses to the request at the end of their interview to briefly review and comment on the ASPPH competencies, gave a high level of agreement that they were relevant to strategic leadership training in Africa. The words of one KI probably explain the high levels of agreement.

‘these are very generic and broad competencies and hard to disagree with’ (KI Ghana).

Some reservations noted by South African respondents were that the proposed competency set is too wide, and that some of the competencies could be seen as programme entry requirements.

### Synthesis

A joint review of the data from the three case studies by consortium partners in Cape Town in February 2015 led to the classification of the identified competencies into three clusters. They are: (1) Building reflective and critically reflexive practice, (2) generating and using evidence and (3) leading change and navigating complex contexts.

Building reflective practice encompasses the elements from the analysis of the KI data that we labelled personal values and character or self mastery. Generating and using evidence encompasses the elements generating, analysing, synthesizing and using evidence and technical skills of relevance to the area in which you lead. Leading change and navigating complex contexts, encompasses the elements visioning, inspiring and leading change, skills for interacting and working with people, navigating complexity and context; political and advocacy and managerial function skills.

### Pan African DrPH programme design

KI views on how a Pan African DrPH programme should be structured included that the success of such a programme should be measured by the difference its graduates make to daily functioning within health systems, and it must be closely linked to daily realities. Not surprisingly, therefore, a dominant theme was that leadership competencies were and should continue to be acquired by a combination of formal training, self-directed learning including extensive reading, mentoring, learning by doing and experience over time. They also spoke about the importance of action learning and research, and continuous opportunities to practice these skills. An effective programme was judged as one that combined this mix of approaches, putting particular emphasis on learning by doing and application of theory to practice.

‘(…) need to have hands on experience of decision making to be a leader’ (KI South Africa).

‘(…) take these students into contexts where leadership is being exercised, take them to either work with the MP, director, minister, the commissioner or somebody for about may be 3–4 months so that they are able to see how those people spend their life, who approaches them for what and introduce them to the world of political decision making, what I call political economy issues’ (KI Uganda).

Formal training was important and had a role to play in helping learners to become conversant with relative theory that was applicable to professional practice. However, it was better to have short periods on campus for learning related to theory as well as peer to peer and peer to facilitator engagement, with longer periods of workplace based learning between.

‘People should stop this funny waste of time bringing people from their jobs to sit here as perpetual students’ KI Ghana.

A South African KI argued that a DrPH must include work placements ‘to do something concrete.’ In addition to academic supervisors, linking trainees to workplace mentors who can support them and encourage self directed learning was also judged important: ‘(…) bring in experts, leaders who are either in the process of those positions or former leaders who have been in these positions to be facilitators basically to share experiences’ (KI Uganda).

The programme vision and mission would need to include building people who were intellectuals, but also able to be analytic, solve problems and make a difference. Training should support the ability to ‘(…) think deep and bring something to the table’ and ‘(…). actually come up with solutions and not just come and listen and not come up with solutions (….)’ (KI Ghana). DrPH training must ‘bring new knowledge into existence, and make a tangible difference’ (KI South Africa).

Potential target groups for the programme were identified as people with existing leadership experience, already in middle and senior level leadership and management positions in the health sector who aspired to move to positions of higher responsibility. Such people could be in the public sector or the private not for profit sector. People in clinical care leadership and management such as Directors, Medical Superintendents and Chief Executives of hospitals were judged to need such training just as much as people in more classical public health positions such as programme managers and directors of health services. One KI also suggested that people working in the private for profit sector in health should also be a target for the DrPH.

In selecting people for the programme, it was important to pay attention to their track record since past history can provide important pointers to people’s inclinations and abilities. This might include proven past willingness to take on some of the roles and tasks already mentioned in the leadership competencies.

A few KI also suggested to consider targeting people who aspire to political leadership positions in health and not only those who aspire to technical leadership.

‘(…) at the end of the day it boils down to politics (…) if you become a deputy minister for health and you are a health professional you’ll have to show leadership in health (…)’ (KI Ghana).

## Discussion

There has been a growth of graduate public health programmes offered in Africa since the 1970 s, particularly in the form of MPH programmes (Daire *et al*. 2014; Erasmus *et al*. 2016). These programmes have provided an invaluable service to the continent, giving more people access to training at an affordable cost and contributed to strengthening the team and operational levels of leadership. Beyond the MPH, formal leadership and management capacity building has been primarily through action-learning within time-bound project based activities and short courses, often offered outside the continent (Conn *et al*. 1996; Daire *et al*. 2014; Reich *et al*. 2016).

Given the earlier major capacity gaps in graduate education available on the continent, it was appropriate to focus on masters programmes. It is not surprising that a terminal degree approach such as the DrPH received almost no attention. However, our observations over the years as health systems practitioners, educators and researchers working in Africa, suggested to us that the need for an African DrPH is now clear and pressing. We judged that it is time for Africa to move to the next stage of expanding leadership capacity building provided at an affordable cost and tailored to context.

We undertook this work for two reasons. First, as a reality check on our judgement–systematically consulting on a larger scale with health system managers and leaders to confirm or disprove our assessment of need. Such work is an essential part of accountability in developing and implementing any such post-graduate programme. It is reassuring to have found such overwhelming support for the concept with the vast majority of our KIs and over 90% of survey respondents in the three countries affirming the need.

Secondly, we wanted to identify the strategic leadership competencies and optimal capacity building approaches in the African context for any such program. Inadequate attention has also been paid to the influence of the context of leadership and the nature of the systems within which leaders operate in sub-Saharan Africa, on the predominant leadership type that arises; and what kind of capacity building empowers leaders to better maneuver within the challenges of context and systems (Kwamie *et al*. 2015). It was important to identify relevant competencies in context to inform program design.

Despite the fact that we set out on an open ended exploration of appropriate competencies in context; the competencies for strategic leadership in Africa that emerged from the analysis of our KI interviews are not very different from what has emerged from work on strategic leadership competencies in other (high income) settings e.g. (Alimo-Metcalfe *et al*. 2008; ASPPH 2009; International Hospital Federation 2015). This does not, however, negate the value of this work. It was important to explore competencies in the low and middle income contexts of the countries in which we are working.

Health systems are complex, adaptive and unpredictable in all settings. We think the competencies that have emerged from this study are similar to those that have emerged in other contexts, because they are a set of principles and skills that enables flexible, rapid and appropriate responses to context specific situations. The way the principles are used and adapted to different situation is what will vary. This would fit with the recurring perception of almost all KIs that strategic leadership capacity development has to be a mix of imparting theory and on the job coaching and mentoring, to support the effective use of theory and principles in context to respond to situations that are often difficult to predict.

Several key next steps are needed to implement a Pan African program, each with its own challenges. One step is ensuring that the design and implementation of a pan-African DrPH program draws upon key findings from the assessment. This includes appropriate selection of candidates, emphasis of critical competency areas and a programme structure that allows for workplace based learning with coaching and mentoring. We envisage that the pan-African DrPH will be targeted primarily at those working in the public health system, given the continuing importance of public leaders as the national stewards of every health system. Experience at team and operational levels of leadership are critical to performance at the strategic level. Therefore, essential in selection considerations will be a minimum of five, preferably more years of leadership and managerial experience at team and operational levels. Additional requirements in selection will be people who are living and working in Africa and intend to continue to do so, and have a demonstrated commitment to achieving public value through health system development on the continent. The latter criterion also allows for those working in non-governmental sectors, e.g. to be considered.

In order to support the development of these competencies, for trainees who will spend most of their time continuing in the workplace, we envisage a 3 to 4 year programme. The first 2 years will be structured around short annual residential periods with considerable time spent learning in the workplace inbetween. The residential periods will allow face-to-face engagement among programme participants and with facilitators to introduce and deepen learning over time around the learning outcomes, through peer to peer as well as peer to facilitator engagement and learning. Trainees will also rotate between the organizations involved to allow exposure to different African settings across the course of the programme. Drawing on the growing body of relevant experience (Doherty and Gilson 2015), workplace based learning activities will, be structured to support the application of the knowledge and practices introduced in the residential periods to workplace activities, and will be supported by virtual and distance learning mechanisms. It is widely recognized that such workplace based learning is essential in leadership development given it requires personal practice development, and identity change, rather than only knowledge acquisition (Lord and Hall 2005). Together the residential periods and workplace-based learning will also lay the foundations for the final dissertation, which will be the focus of the last 2 years of the program. This will entail the implementation of a larger-scale systematic inquiry into a workplace relevant topic, with an emphasis on practice-oriented, action research, reflective practice and implementation science projects. Such inquiry might take the form of an intervention evaluation, a policy or implementation analysis, an organizational assessment or an analysis using routine data to inform a key decision, e.g. Supervision would ideally be provided by an academic supervisor working with a practice supervisor, that is an experienced health system leader.

We propose a 3 to 4 year course as the ideal length for a professional terminal degree for two reasons. Firstly, from the assessment the program has to be work place based and not a full time residential programme, to enable the large work place based learning component that is an essential part of its applied nature. Secondly, in all the tertiary institutions, 3 to 4 years is the standard requirement for a terminal degree.

Some have suggested that a shorter, Master’s level leadership qualification is sufficient to meet the needs of the target audience. It might, then, be possible to offer a master’s programme for those completing the first 2 years, with the addition analytic and synthetic work of the full 4 year progamme clearly providing the basis for a terminal degree award. The terminal degree option would be especially valuable for those who are interested in professional health sector leadership, as well as those interested in teaching, coaching and mentoring the next generation of public health leaders. Whether those who complete a terminal degree perform better in leadership than those who complete an executive masters is a question that would need a longitudinal research design.

Programme affordability for trainees must also be considered. 2017/18 proposed fees for non-Ghanaian nationals registered full time for a doctoral program in the College of Health Sciences of the University of Ghana are US$36 364 over 4 years (about US$9000 per annum). Fees for Ghanaian nationals are about half this amount (about US$4500 per annum) because of government subsidies (University of Ghana 2017). It will be important to explore options for part or full scholarships and subsidies from governments and employers. The stakeholder consultative approach that has been used for this assessment and the development of competencies provides a base for continuing discussions with employers and other potential sponsors.

Importantly, however, at these rates, the costs of a programme based in Africa are lower than current DrPH programmes in the USA and the UK. For example,

- some 2016/17 academic year fees: non-resident tuition fees for the UNC-Chapel Hill School of Public Health Executive Doctoral program were US$1545.42 per credit hour (http://sph.unc.edu/hpm/hpm-drph-tuition-fees/). Given the 45–51 credit hours needed to graduate tuition fees were about US$69 543—78 816 over the 4 years of the program (about US$15 000–20 000 per annum), for the 45 - 51 credit hours required to graduate
- John Hopkins Bloomberg School of Public Health, DrPH programme fees were US$52 368 per academic year for full time enrollees and US$1091 per credit hour for part time enrollees (http://www.jhsph.edu/admissions/tuition-and-fees/),
- Harvard T. Chan School of Public Health, DrPH programme full time tuition fees were US$42 880/year and part time fees US$21 440/year (https://www.hsph.harvard.edu/admissions/admissions/tuition/),
- London School of Hygiene and Tropical Medicine, DrPH programmes fees were GBP 14,450 for full time students and GBP 7,225 for part time students in 2015/16. (https://www.lshtm.ac.uk/study/fees-funding/tuition-fees/tuition-fees-and-expenses-2016-17).

Another important consideration in moving to programme implementation is that although tertiary academic institutions in Africa have, to varying degrees, capacity assets such as ongoing teaching and research, staff, infrastructure etc., these are generally very inadequate in relation to needs. Undergraduate and post graduate training institutions are challenged by contextual, economic, human and other resource and capacity constraints (Kabiru *et al*. 2010; Mirzoev et al. 2013; Kabiru *et al*. 2014; Agyepong *et al*. 2015). The strategy proposed by the Pan African DrPH consortium to work round these challenges includes pooling of resources through shared, open access curriculum, as well as faculty and student exchanges and the development of shared distance learning options.

Finally, navigating the proposals of this pan-African DrPH concept through the organizational waters of partner universities’ accreditation systems will be a further challenge. This can be a very lengthy process. We hope that this study will both provide support for this effort and offer a baseline against which to assess it in future.

## Conclusions

This study has provided information about perceptions of need and competencies, program content and structure for a Pan African DrPH programme with a focus on strategic leadership capacity building to support public health, health systems strengthening and ultimately health outcome improvement in Africa. Though support for such a program is not universal, it would appear to be large enough to merit the continued development and implementation as well as monitoring and evaluation of the processes and impact of such a program.
